# Brain-wide fMRI responses to auditory and visual stimuli in awake mice

**DOI:** 10.1093/cercor/bhag137

**Published:** 2026-09-16

**Authors:** Jaakko Paasonen, Lenka Dvořáková, Petteri Stenroos, Raimo A Salo, Ekaterina Paasonen, Shalom Michaeli, Silvia Mangia, Heikki Tanila, Olli Gröhn

**Affiliations:** A. I. Virtanen Institute for Molecular Sciences, University of Eastern Finland, Neulaniementie 2, 70210 Kuopio, Finland; A. I. Virtanen Institute for Molecular Sciences, University of Eastern Finland, Neulaniementie 2, 70210 Kuopio, Finland; A. I. Virtanen Institute for Molecular Sciences, University of Eastern Finland, Neulaniementie 2, 70210 Kuopio, Finland; A. I. Virtanen Institute for Molecular Sciences, University of Eastern Finland, Neulaniementie 2, 70210 Kuopio, Finland; A. I. Virtanen Institute for Molecular Sciences, University of Eastern Finland, Neulaniementie 2, 70210 Kuopio, Finland; NeuroCenter, Kuopio University Hospital, Puijonlaaksontie 2, 70210, Kuopio, Finland; Center for Magnetic Resonance Research, University of Minnesota, 2021 Sixth Street SE, Minneapolis, MN 55455, United States; Center for Magnetic Resonance Research, University of Minnesota, 2021 Sixth Street SE, Minneapolis, MN 55455, United States; A. I. Virtanen Institute for Molecular Sciences, University of Eastern Finland, Neulaniementie 2, 70210 Kuopio, Finland; A. I. Virtanen Institute for Molecular Sciences, University of Eastern Finland, Neulaniementie 2, 70210 Kuopio, Finland

**Keywords:** anesthesia, auditory, awake, functional magnetic resonance imaging, mouse, stimulation, visual, zero echo time

## Abstract

The functions of individual senses are well studied, but the mechanisms of cross-sensory integration remain poorly understood. Although invasive studies in mice can elucidate cellular and circuit-level processes, it is unclear whether mice exhibit macroscale cross-sensory activity comparable to that reported in human imaging studies—an important criterion for the translational relevance of preclinical studies. To address this gap, we investigated responses to unimodal and bimodal auditory and visual stimulation in awake mice using a quiet zero echo time (zero-TE) functional magnetic resonance imaging (fMRI) approach. Both stimulation modalities evoked responses in cross-sensory primary pathways, high-order regions, non-primary thalamus, and cerebellum, indicating robust brain-wide responses to simple sensory stimulation. Bimodal stimulation elicited subadditive responses in regions responsive to both modalities, and primary cortices contained subregions with stronger cross-modal responses. Many of these patterns resembled those observed in humans and non-human primates, suggesting conserved principles across species and supporting the translational value of mechanistic and disease model-oriented studies of cross-sensory processing in mice. In contrast, ketamine-xylazine anesthesia caused widespread suppression of cross-modal and high-order activity during sensory processing, highlighting the importance of performing cross-sensory investigations in awake animals.

## Introduction

Sensory systems have been long studied as independent units, but accumulating evidence indicates that many thalamic and cortical regions once considered sense-specific respond to multiple sensory modalities (Ghazanfar and Schroeder 2006; Driver and Noesselt 2008; Stein and Stanford 2008). Because of the relatively recent attention, far less is understood about the cross-sensory integration processes, compared with the detailed understanding of individual senses (Opoku-Baah et al. 2021). By integrating early sensory input from multiple sources, the brain accelerates the encoding, decoding, and interpretation of information to better characterize natural events and objects (Stein and Stanford 2008).

In addition to its fundamental neurobiological importance, understanding cross-sensory integration has a broad clinical relevance, as impaired integration of sensory information has been implicated in several central nervous system disorders, including autism spectrum disorder (Beker et al. 2018), attention-deficit/hyperactivity disorder (Schulze et al. 2023), Alzheimer’s disease (Hong et al. 2024), neurodevelopmental dyslexia (Francisco et al. 2017), schizophrenia (Tang et al. 2025), and sensorineural hearing loss (Dai et al. 2018). Therefore, a deeper understanding of the neurophysiological cascade underlying multisensory integration can, in turn, facilitate diagnosis, drug development, and therapeutic intervention for these conditions (Opoku-Baah et al. 2021).

Non-invasive techniques such as electroencephalography, magnetoencephalography, and functional magnetic resonance imaging (fMRI) have long been used to characterize the functions of individual sensory systems, yielding a detailed understanding of sensory processing in numerous preclinical studies (Cheung et al. 2012; Lau et al. 2013; Gao et al. 2015; Toarmino et al. 2017; Sanganahalli et al. 2022; Gil et al. 2024, 2025; Severo et al. 2025; Carvalho et al. 2026). Neuroimaging studies have also provided initial insights into when and where cross-sensory activity and multisensory integration occur in the human brain (Ghazanfar and Schroeder 2006; Driver and Noesselt 2008; Stein and Stanford 2008; Petro et al. 2017; Opoku-Baah et al. 2021; Choi et al. 2023). Comparable imaging studies in animals, however, remain scarce, with only a few in non-human primates (Kayser et al. 2007; Guipponi et al. 2015) and rodents (Dinh et al. 2024; Paasonen et al. 2025). Preclinical mechanistic experiments are crucial, since invasive techniques are necessary to disentangle multisensory integration processes at the level of cortical layers or even single neurons (Leong et al. 2018). Ideally, one would measure behavior, macroscale brain activity, and cell-level electrical activity simultaneously. This is technically feasible due to the recent advances in preclinical imaging techniques for rodents (Paasonen et al. 2020, 2022; Dvořáková et al. 2024), making such investigations highly attractive for sensory research. Nevertheless, it remains unclear whether similar cross-sensory brain activity can be detected with neuroimaging methods in rodents and humans, which would provide the foundation for translational cross-sensory research.

Therefore, the primary aim of this study was to investigate cross-sensory and high-order brain activity in awake mice using fMRI and to compare these findings with those reported in humans. To facilitate cross-species comparisons, we employed unimodal auditory and visual stimuli, which are the most commonly used modalities in human cross-sensory studies (Calvert 2001; Laurienti et al. 2002; Johnson and Zatorre 2005; Baier et al. 2006; Ghazanfar and Schroeder 2006; Martuzzi et al. 2007; Raij et al. 2010; Hsieh et al. 2012; Gu et al. 2020; Opoku-Baah et al. 2021; Choi et al. 2023). Imaging was performed with a zero echo time (zero-TE) fMRI (Lehto et al. 2017; Paasonen et al. 2020), as it provides a good proxy for brain electrical activity (Valjakka et al. 2025), is well-suited for awake measurements due to its resistance to movement-induced artifacts (Paasonen et al. 2022; Dvořáková et al. 2024; Mackinnon et al. 2025), and due to its silence (Paasonen et al. 2020; Dvořáková et al. 2024; Mackinnon et al. 2025). Quiet operation is especially critical because the loud acoustic noise generated by conventional fMRI pulse sequences may confound the detection of auditory-related cross-sensory processing (Laurienti et al. 2002; Martuzzi et al. 2007; Raij et al. 2010; Cheung et al. 2012; Toarmino et al. 2017). Secondary aims were to assess whether bimodal auditory and visual stimulation evokes interactions in the mouse brain comparable to those observed in humans, and to examine whether ketamine-xylazine anesthesia, commonly used in preclinical imaging with mice, acts as a confounding factor in cross-sensory processing.

For clarity, terms used in this report are defined as follows: “cross-sensory” refers to activity in non-primary regions evoked by input to a primary sensory system (e.g. auditory-evoked activity in visual cortex); “unisensory” and “unimodal” refer to events or activity involving a single sensory modality, allowing straightforward evaluation of cross-sensory characteristics; and “multisensory” and “multimodal” refer to the involvement of two or more sensory modalities.

## Materials and methods

The present study reanalyzes previously published data (Dvořáková et al. 2024) to examine brain-wide cross-sensory activity during unisensory auditory and visual stimulation and responses to concurrent stimulation, which were not analyzed previously. As most methods were described in detail previously (Dvořáková et al. 2024), only a brief summary is provided here, together with the additional relevant methodological details. Group-level values are reported as mean ± standard deviation unless otherwise specified.

### Animals

Animal procedures were approved by the National Animal Experiment Board (licenses ESAVI-2023-19957OH3 and ESAVI­2019­028408) and conducted in accordance with the European Commission Directive 2010/63/EU guidelines. A total of eight adult (four males and four females; 8–12 months) C57BL/6 mice (Lab Animal Centre, University of Eastern Finland, Kuopio) were used across 43 fMRI sessions (20 under anesthesia, 23 in the awake state). Mice were individually housed to minimize damage to the head-implant and surgical wound and maintained on a 12/12 h light–dark cycle at 22 ± 2 °C with 50% to 60% humidity. Food and water were available ad libitum. All experiments were carried out between 8 a.m. and 5 p.m.

### Surgery for chronic head-fixation implant

To minimize skull movement during fMRI, mice underwent surgery for chronic head-fixation implant placement. Mice were anesthetized with isoflurane (Attane vet 1000 mg/g, Piramal Healthcare UK Limited, Northumberland, UK; 5% for induction, 1.8%–2.2% for maintenance) in 30/70% O_2_/N_2_ carrier gas mixture. After exposing and cleaning the skull, a headpost made of either polytetrafluoroethylene or polychlorotrifluoroethylene was affixed using dental cement (Selectraplus, DeguDent GmbH, Hanau, Germany). Carprofen (5 mg/kg s.c.; Rimadyl, Zoetis Finland Oy, Helsinki, Finland) was administered for analgesia. Following surgery, mice were allowed to recover for at least 3 weeks before the habituation protocol began.

### Awake imaging protocol

The mice were gradually acclimatized to imaging to minimize movement and stress. The 14-day habituation protocol (Dvořáková et al. 2024), similar to our previous protocols (Stenroos et al. 2018; Paasonen et al. 2020, 2022; Dvořáková et al. 2022), was distributed over 3 weeks. During the first 5 days, animals were allowed to familiarize themselves with the handler, imaging holder, and sounds of the MRI scanner. Additionally, the headpost was manually held still for short periods of time. Over the following 9 days, mice were lightly anesthetized with isoflurane (5% for induction, 1.6–2.0% for maintenance in N_2_/O_2_ 70%/30% mixture) and head-fixed to the imaging holder, while the rest of the body remained free to move. Ear plugs were also introduced. Mice were then allowed to awaken in the holder and were exposed to the recorded sound of the fMRI sequence. The habituation time was gradually increased from 5 to 25 min. During the final 3 days, habituation also included auditory and visual stimuli presented with a similar pattern as during fMRI. A positive reward (1% sucrose water or sweetened hazelnut cocoa spread) was given before and after each habituation and measurement session.

For fMRI experiments, the procedures were similar to the habituation protocol. Isoflurane anesthesia was maintained slightly longer to allow for additional preparations, including radiofrequency coil placement, imaging preparations, and anatomical imaging. After acquiring anatomical images, isoflurane was discontinued, and fMRI began 2–3 min later, once mice exhibited clear signs of consciousness, such as limb, jaw, or whisker movement. The first stimulus was delivered 5 min after the start of fMRI acquisition, allowing further stabilization of physiological condition. During fMRI, breathing rate (113 ± 29 breaths per minute) and behavior were monitored using an MRI-compatible video camera (12 M-i, MRC Systems GmbH, Heidelberg, Germany). After the fMRI measurement, mice were briefly re-anesthetized with isoflurane before being released from the holder and returned to their cages.

### Ketamine-xylazine anesthesia protocol

For fMRI measurements under ketamine-xylazine anesthesia, mice were initially anesthetized with isoflurane (3% in N_2_/O_2_ 70%/30% mixture). A single intraperitoneal injection of ketamine (Ketaminol Vet 50 mg/ml, Intervet International B.V, Boxmeer, Netherlands; 100 mg/kg) and xylazine (Rompun Vet 20 mg/ml, Bayer Animal Health GmbH, Leverkusen, Germany; 10 mg/kg) was then administered (6.7 mL/kg). Following injection, isoflurane was gradually reduced and maintained at 0.5% while ear plugs were inserted and the animal was positioned in the holder. Isoflurane was discontinued typically 15–30 min after the initial induction. Body temperature (∼37 °C) and respiration (214 ± 26 breaths per minute) were monitored with MRI-compatible equipment (Model 1025, Small Animal Instruments Inc., New York, NY, USA), and mice were kept warm with water circulation system (Corio CD, Julabo, Seelbach, Germany). After the experiments, mice were returned to their cages and placed on a heated surface to ensure maintenance of normal body temperature during recovery.

### Auditory and visual stimulation

The stimulation setups and parameters are schematically illustrated in Supplementary Fig. 1. During fMRI, auditory, visual, or combined stimuli were presented for 10 s with 1-min intervals. Each stimulus type was repeated 5 times, resulting in a total of 15 stimuli per measurement. The order of stimulus presentation was randomized for each experiment. Stimuli were controlled using a stimulus generator (STG4008–16 mA, Multi-Channel Systems MCS GmbH, Reutlingen, Germany) controlled by MC_Stimulus II software (V 3.5.11). The first stimulus was delivered 5 min after the start of fMRI acquisition.

For auditory stimulation, pressurized air (2 bar) was gated by a TTL signal-controlled solenoid valve (Neos Biotec, Pamplona, Spain). Air puffs (5 ms) were delivered at 13 Hz through a plastic tube positioned at the midline of the animal holder, ~15 cm posterior to both ears of the mouse (Supplementary Fig. 1). The average sound pressure levels, measured as previously described (Paasonen et al. 2020), at the level of animal inside the scanner were 56 dB, 69 dB, and 82 dB during baseline conditions, during fMRI sequence, and during fMRI sequence with auditory stimulation, respectively. Bench tests indicated that the power spectrum of sound resembled white noise.

For visual stimulation, a custom-built TTL signal-controlled, Arduino-based light emitter was used. Blue light flashes (470 nm, 50 ms) were delivered at 5 Hz through optical fibers positioned to illuminate both eyes directly, without a diffuser or projection screen (Supplementary Fig. 1). The light intensity, measured on the bench at a distance of 5 mm from a single optical fiber, was 41 lx (PeakTech 5035, Ahrensburg, Germany).

### Magnetic resonance imaging

MRI was performed using a 9.4 T, 31-cm bore magnet with a 12-cm inner diameter gradient coil set, interfaced with an Agilent DirectDRIVE console (Palo Alto, CA, USA). A linear radiofrequency surface coil (11 × 13 mm inner diameter loop; Neos Biotec, Pamplona, Spain), fitting around the headpost, was used for both transmission and reception.

Anatomical images were acquired with a zero-TE 3D radial Multi-Band SWeep Imaging with Fourier Transformation (MB-SWIFT) sequence (Idiyatullin et al. 2015) using the following parameters: 4,000 spokes per spiral, 16 stacks of spirals, repetition time ∼3 ms per spoke, four radiofrequency pulses per spoke, flip angle 4°, field of view bandwidth 192 kHz, oversampling factor 2, matrix size 256^3^, field of view 24^3^ mm, and 94 μm isotropic resolution. To enhance anatomical contrast, a magnetization transfer pulse (γB1 = 125 Hz, offset = 2000 Hz, duration = 20 ms) was applied every 32 spokes. The total acquisition time was ∼4 min.

Functional images were also acquired with the MB-SWIFT sequence with the following parameters: 2,047 spokes per spiral, one spiral, repetition time 0.82 ms per spoke, two radiofrequency pulses per spoke, flip angle 3°, field of view bandwidth 125 kHz, oversampling factor 4, matrix size 64^3^, field of view 24^3^ mm, and 375 μm isotropic resolution. The acquisition time for a single fMRI volume was ∼1.7 s. Total of 710 volumes (∼20 min) were acquired in each fMRI scan. The 15-min stimulation paradigm was preceded by a 5-min task-free period to ensure stabilization of baseline signals and physiological state. A representative fMRI image, along with the corresponding anatomical reference template, is shown in Supplementary Fig. 2.

### Data processing and analysis

The MB-SWIFT raw data were reconstructed into NIfTI format on a volume-by-volume basis using radiofrequency pulse deconvolution, center-out data re-gridding, and the iterative FISTA algorithm (Beck and Teboulle 2009) with three iterations. To minimize potential effects of drift and motion, the fMRI data were motion-corrected using rigid co-registration of each 3D volume to the first volume with Advanced Normalization Tools (ANTs, http://stnava.github.io/ANTs). The six motion-correction parameters were saved for later use as regressors. DVARS (root mean square of the intensity difference between consecutive volumes) values were 19.6 ± 2.6 in anesthetized animals and 23.5 ± 3.3 in awake animals, resulting in 0.1% (anesthetized) and 5.9% (awake) of volumes being flagged as potentially containing motion-related artifacts using a threshold of 30 (see more details and discussion in (Dvořáková et al. 2024)).

The reconstructed and masked anatomical brain volume from each session was then co-registered to a reference template, selected from among the datasets, using affine and nonlinear SyN registration in ANTs. Brain masks were generated with a modified version of Medic-DeepLabv3+ (Valverde et al. 2023). To achieve accurate co-registration of the low-resolution, low-contrast fMRI data, the linear and non-linear transformation parameters from the anatomical co-registration were applied to the corresponding fMRI datasets. No spatial smoothing or global signal regression was applied.

Experiment-level statistical activation maps for three stimulus types (auditory, visual, and combined) were calculated using a general linear model-based FLAME approach implemented in the FSL FEAT toolbox (version 6.0; https://fsl.fmrib.ox.ac.uk/fsl/). Data were high-pass filtered at 0.01 Hz, and prewhitening was performed using FSL FEAT’s default AR(1) autocorrelation model. A whole-brain mask was applied. The six motion-correction parameters were included as regressors. Hemodynamic response function parameters were set as follows: delay, 2.8 s; dispersion, 1.4 s. Stimulus duration was set to 10 s for the main results and 2 s for supplementary analyses to investigate sharper responses. Significant responses in group-level statistical maps were assessed using permutation-based inference implemented in Permutation Analysis of Linear Models (PALM in FSL), with multiple comparisons controlled using family-wise error (FWE) correction with *P* < 0.01. For supplementary qualitative analysis of individual- and sex-specific maps, a threshold of t-value > 2 was used.

Regions-of-interest (ROI) were drawn with FSLeyes on the template brain according to an anatomical atlas (Franklin and Paxinos 2007). Regions showing significant signal changes (*P* < 0.01, FWE-corrected) in any of the group-level maps were included in the analysis. Prior computing average time series from each ROI, the motion-correction parameters were regressed out voxel-wise. Average time series across all subjects were then calculated for each selected region, and a slight temporal smoothing was applied. The average response values for each ROI were calculated from a 10-volume (17-s) window starting at the stimulus onset. Within the same window, one-sample *t-*tests were performed to assess whether the response during each volume differed from zero. For average responses, we tested whether differences exist between awake and anesthetized mice (two-sample *t-*test). We also examined whether simultaneous auditory and visual stimulation elicited significant interactions in the region-specific response by subtracting the individual auditory and visual responses from the combined response and testing whether this difference deviated from zero (one-sample *t-*test).

Where applicable, p-values were corrected for multiple comparisons (either FWE or Bonferroni correction, as specified) and values < 0.05 were considered statistically significant, unless stated otherwise.

## Results

### Robust cross-sensory and high-order responses to auditory and visual stimuli in awake mice

**Figure 1:**
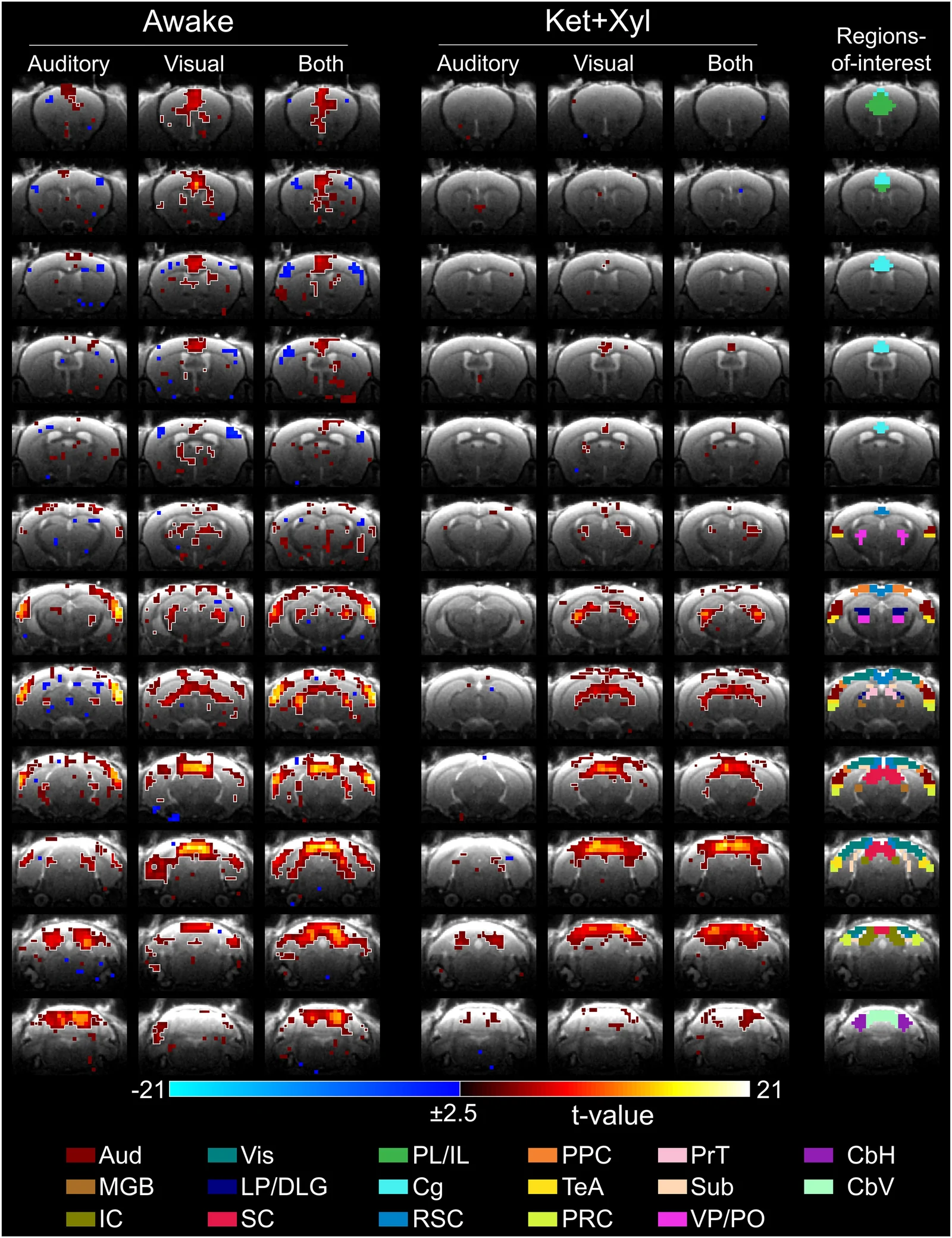
Activation maps in response to auditory, visual, and bimodal stimuli in awake and anesthetized mice, along with regions-of-interest derived from the activated regions. White outlines on the maps indicate regions with *P* < 0.01 (FWE-corrected), and color maps are thresholded at t > 2.5. Results are based on 100–115 stimulus blocks collected across 20–23 scans per group. Every other coronal slice is shown, overlaid on an anatomical reference image. Aud, auditory cortex; CbH, cerebellar hemisphere; CbV, cerebellar vermis; cg, cingulate cortex; DLG, dorsal lateral geniculate nuclei; IC, inferior colliculus; IL, infralimbic cortex; Ket+Xyl, ketamine-xylazine anesthesia; LP, lateral posterior thalamic nuclei; MGB, medial geniculate body; PL, prelimbic cortex; Po, posterior thalamic complex; PPC, posterior parietal cortex; PRC, perirhinal cortex; PrT, pretectal area; RSC, retrosplenial cortex; SC, superior colliculus; sub, subicular complex; TeA, temporal association cortex; Vis, visual cortex; VP, ventral posterior thalamic complex.

Figure 1 summarizes the group-level statistical activation maps for auditory, visual, and combined stimuli, while Supplementary Fig. 3 and Supplementary Video 1 show 3D presentations of the significant clusters (*P* < 0.01, FWE-corrected). As reported previously (Dvořáková et al. 2024), auditory stimulation elicited robust responses in auditory tracts, including the inferior colliculus (IC), medial geniculate complex (MGB), and auditory cortex (Aud) in awake mice, whereas responses were restricted to the IC under anesthesia. Visual stimulation induced robust responses in visual tracts, including the superior colliculus (SC), lateral posterior nucleus (LP), dorsal lateral geniculate nucleus (DLG), pretectal area (PRT), and visual cortex (Vis) in both groups. These observations confirm that both primary sensory tracts were successfully stimulated, and that the ketamine-xylazine anesthesia severely confounds primary auditory processing.

In addition to the traditionally expected findings, we observed significant signal changes in cross-sensory cortices in awake mice: auditory stimulation elicited responses in Vis, while visual stimulation elicited responses in Aud (Fig. 1). These cross-sensory responses were absent under anesthesia. In addition, awake mice showed robust responses to visual stimulation in several high-order regions, including cingulate cortex (Cg), pre- and infralimbic cortex (PL/IL), perirhinal cortex (PRC), temporal association cortex (TeA), posterior parietal cortex (PPC), retrosplenial cortex (RSC), and subicular complex (Sub). Auditory stimulation engaged many of these regions, though in a more restricted manner. A few voxels (t > 2.5) extended into the motor cortices, but due to their small number they were omitted from subsequent analyses. Under anesthesia, auditory stimulation did not evoke responses in any high-order regions, whereas visual stimulation produced limited activity in areas such as RSC and Sub. Taken together, a robust and widespread cross-sensory and high-order activity was detected in awake mice, in contrast to anesthetized mice.

In addition to primary and high-order areas, awake mice showed responses in thalamic regions encompassing the ventral posterior (VP) and posterior (Po) complex to both stimulation modalities (Fig. 1). Under anesthesia, visual stimulation elicited responses in the same regions, while no clear responses were detected to auditory stimulation. In the cerebellum, the vermis of lobules 3–5 (CbV) responded primarily to auditory stimulation, while the hemisphere of lobule 6 (CbH) also responded to visual stimulation in awake mice. In anesthetized mice, responses in these cerebellar regions were visible but more restricted. These findings indicate that responses in non-primary thalamic and cerebellar regions can be detected in both awake and anesthetized mice, yet they depend on the stimulation modality.

To assess how consistently the above-described activation patterns were expressed at the individual level, voxel-level incidence maps were generated from individual-level maps (Supplementary Fig. 4). While sense-specific responses in primary sensory areas were observed in nearly all individuals, many cross-sensory and higher-order responses were visible in only 40%–60% of the individual-level maps. This likely reflects the smaller signal amplitudes of the cross-sensory responses, emphasizing the need for larger datasets to reliably detect these effects. Additionally, incidence maps were calculated separately for male and female mice (Supplementary Fig. 5). The resulting maps closely resembled one another, suggesting no clear sex-dependent effects.

As the awake imaging experiments were preceded by a brief period of isoflurane anesthesia, we investigated whether any consistent differences in spatial activation patterns were present between the first five and last five stimulation blocks, which could indicate lingering effects of anesthesia. We found no significant differences between the two datasets for unimodal stimuli and only a few scattered voxels for bimodal stimulation (Supplementary Fig. 6). These findings provide no clear evidence that acute anesthesia effects substantially influenced the spatial distribution of the observed responses.

Although clusters with negative t-values were present in some regions of the primary sensory and motor cortices in awake animals (Fig. 1), these did not exceed the selected significance threshold in the current work (*P* < 0.01, FWE-corrected). Therefore, the analyses focused on positive responses.

### Bimodal stimulation can both spatially expand and restrict the regional responses compared to unimodal stimulation

We selected 17 anatomical ROIs for subsequent analyses based on the observed responses (Fig. 1, Supplementary Fig. 3B, and Supplementary Video 1). These ROIs are listed in Table 1, which also shows the overlap of each activation map with the corresponding ROI. Although the localization of significant signal changes to combined stimulation appeared to closely resemble the concatenation of corresponding unimodal maps (Fig. 1 and Supplementary Fig. 3A), with no clear evidence of additional areas activated, the spatial overlap proportions indicate that in regions such Aud, Vis, TeA, PRC, PPC, MGB, Sub, and CbH, the response to bimodal stimulation in awake mice covered a larger area than the response to unimodal stimulus (Table 1). This may reflect the presence of modality-specific intra-regional processing zones. Conversely, in other regions, such as Cg, RSC, LP/DLG, VP/Po, and PrT, the responses were spatially more restricted under bimodal stimulation (Table 1), suggesting potential inhibitory interactions.

**Table 1:** Regions-of-interest (ROIs) derived from the statistical maps. ROIs were grouped into five categories, as shown on the left. The percentages on the right indicate the spatial overlap of voxels in the statistical maps (*P* < 0.01, FWE-corrected) for each ROI. The size of each ROI is shown in the middle. Ket+Xyl, ketamine-xylazine anesthesia.

				Spatial overlap of statistically significant voxels (*P* < 0.01, FWE-corrected)					
				Awake			Ket+Xyl		
	Region-of-interest	Abbreviation	Size (voxels)	Auditory stim.	Visual stim.	Both	Auditory stim.	Visual stim.	Both
Auditory tracts	Auditory cortex	Aud	145	88%	30%	93%	0%	0%	0%
	Auditory thalamic nuclei: Medial geniculate complex	MGB	26	62%	8%	77%	4%	0%	12%
	Inferior colliculus	IC	128	94%	19%	95%	48%	36%	75%
Visual tracts	Visual cortex	Vis	203	24%	31%	48%	0%	74%	62%
	Visual thalamic nuclei: Lateral posterior nucleus and dorsal lateral geniculate nuclei	LP/DLG	38	5%	79%	68%	0%	84%	82%
	Superior colliculus	SC	135	6%	77%	75%	1%	81%	84%
High-order regions	Cingulate cortex	Cg	96	53%	66%	89%	0%	16%	13%
	Orbitofrontal cortex: Orbital, prelimbic, and infralimbic cortex	OFC	98	78%	49%	84%	0%	8%	3%
	Parahippocampal cortex	PHC	65	42%	46%	72%	0%	17%	9%
	Parietal association cortex	PtA	62	4%	80%	82%	0%	0%	0%
	Retrosplenial cortex	RSC	101	5%	88%	80%	0%	29%	0%
	Temporal association cortex	TeA	80	5%	52%	45%	2%	69%	53%
Polymodal thalamic areas	Multisensory nuclei: Ventral posteromedial, ventral posterolateral, and posterior complex	VPM/VPL/Po	68	0%	67%	58%	0%	75%	75%
	Pretectal area	PRT	24	7%	43%	19%	0%	34%	31%
	Subiculum	Sub	48	13%	56%	63%	4%	75%	69%
Cerebellum	Hemisphere of lobule 6 (simplex)	H6	68	77%	3%	77%	0%	0%	11%
	Vermis of lobules 4 and 5	V4–5	35	59%	19%	66%	13%	29%	35%

Some similar observations were made in anesthetized mice. Despite generally weak to no responses to auditory stimulation in auditory pathways, visual stimulation appeared to markedly expand the spatial responses in MGB and IC (Table 1), suggesting cross-sensory input from the visual pathways to auditory regions in both awake and anesthetized conditions. However, in contrast to awake mice, auditory stimulation under anesthesia generally had a restrictive effect on spatial coverage of the responses, such as in PL/IL, Vis, TeA, PRC, RSC, and Sub, the relative response coverage decreased markedly during bimodal stimulation compared to visual stimulation alone (Table 1). Thus, the confounding effect of ketamine-xylazine anesthesia on auditory processing may extend broadly to sensory integration processing as well.

### Awake mice exhibit high response amplitudes and distinct cross-sensory temporal profiles

**Figure 2:**
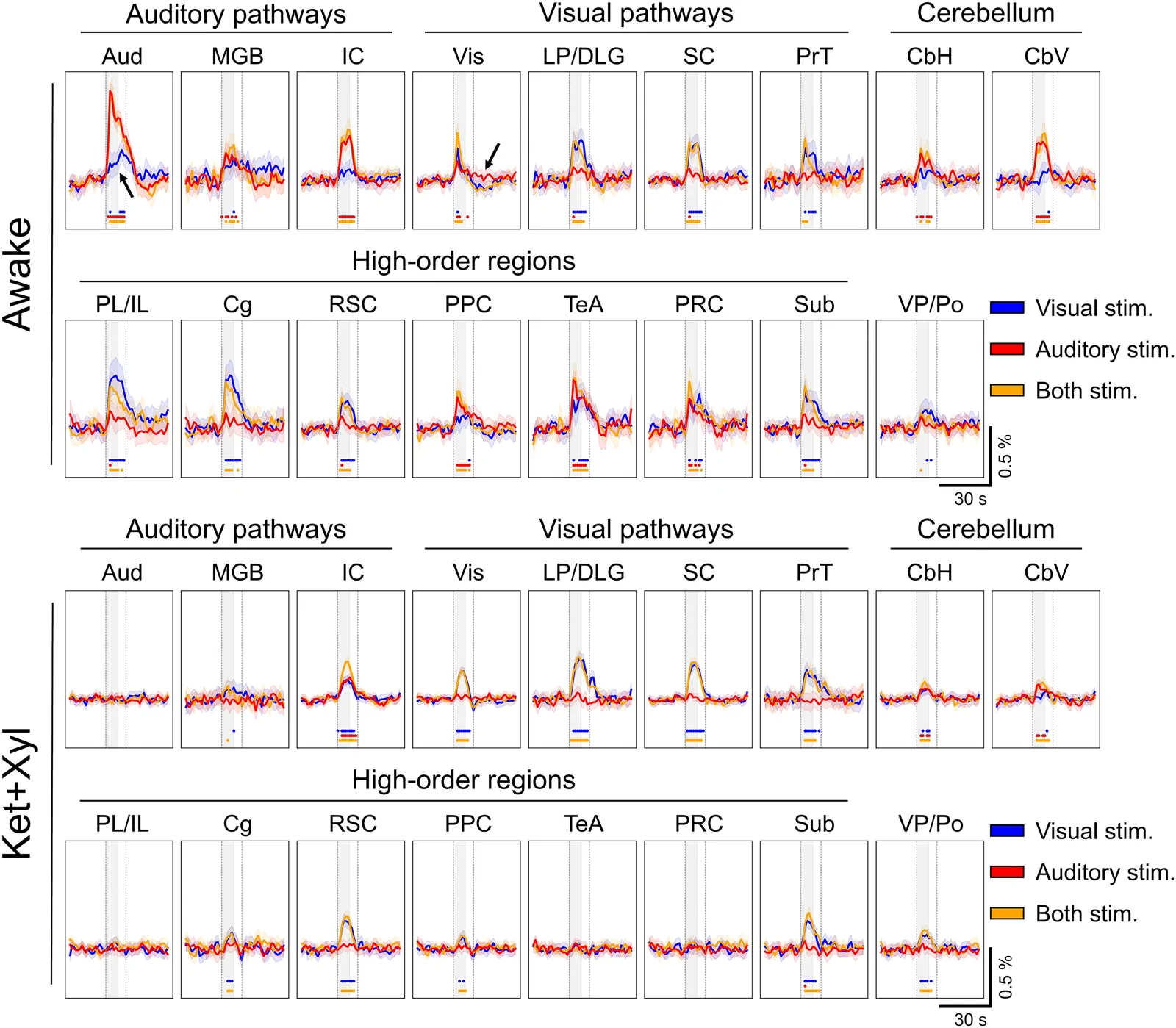
Group-level mean time series from the regions-of-interest (ROIs) in both groups. Each mean time series is averaged across 100–115 stimuli. The shaded vertical gray region marks the 10-s stimulus block. Gray lines indicate the window for calculating average responses. Colored dots below the time series denote time points that differ significantly from zero (*P* < 0.05, Bonferroni-corrected; one-sample *t-*test). Shaded areas around the mean curves represent 90% confidence interval. Black arrows highlight the differing temporal characteristics between responses to primary and cross-sensory stimuli. Abbreviations for ROIs are provided in Fig. 1 and in Table 1. Ket+Xyl, ketamine-xylazine anesthesia.

**Figure 3:**
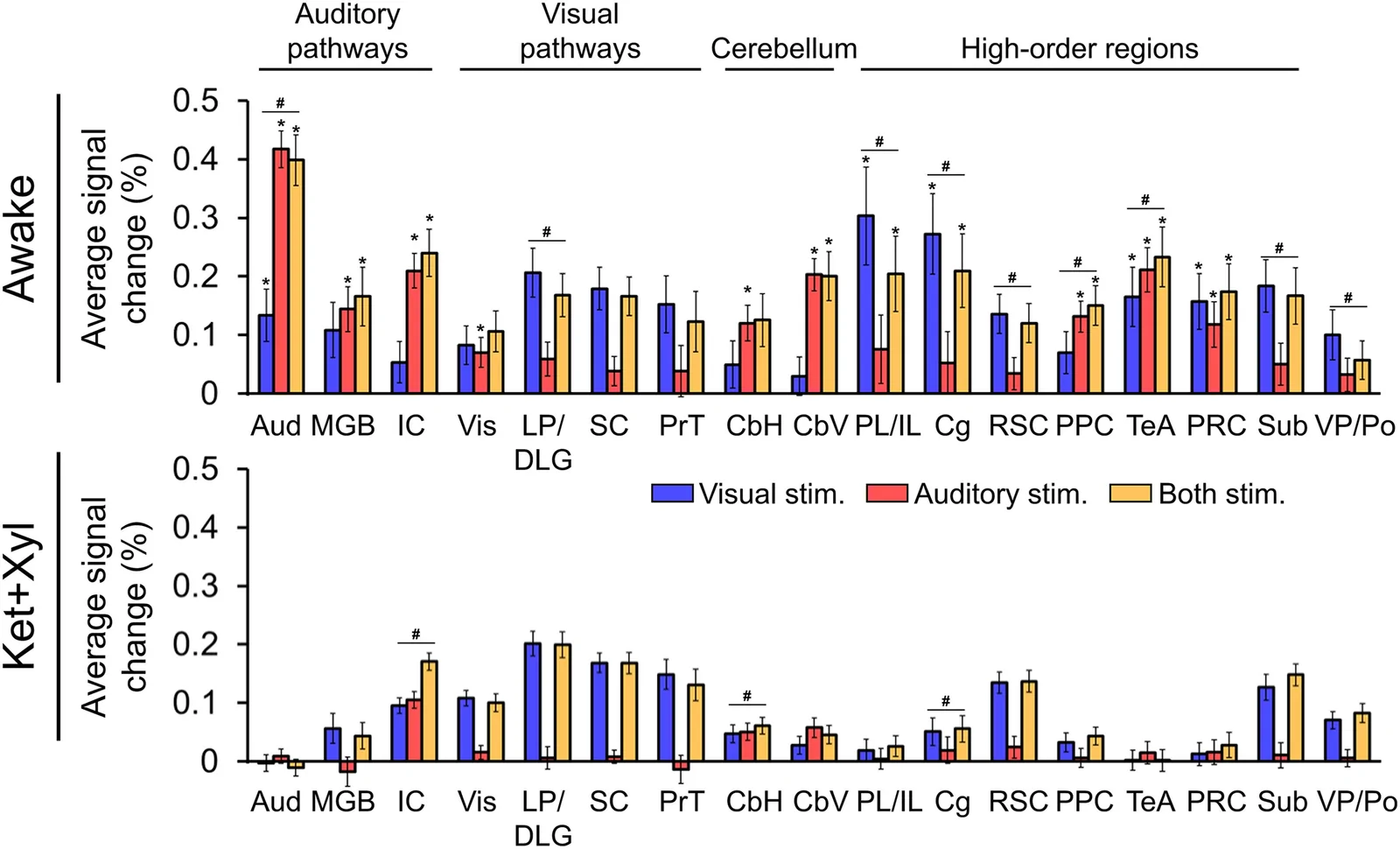
Group-level average fMRI response in each region-of-interest (ROI) for both groups. Average responses were calculated over a 10-volume window beginning at stimulus onset. Each bar represents the mean of 100–115 stimuli. Asterisks (*) indicate differences in response amplitude (*P* < 0.05, Bonferroni-corrected; two-sample *t-*test) between awake and anesthetized mice. To assess interaction effects, we tested whether the response to bimodal stimulation differed from the sum of the unimodal auditory and visual responses. Hashes (#) denote interactions (*P* < 0.05, uncorrected; one-sample *t-*test). Values related to the interaction tests are provided in Supplementary Table 1. Error bars represent the 90% confidence interval. ROI abbreviations are given in Fig. 1 and in Table 1. Ket+Xyl, ketamine-xylazine anesthesia.

Group-level average time series and response amplitudes are shown in Fig. 2 and Fig. 3, respectively. Consistent with Fig. 1, responses in cross-sensory cortices and high-order regions, as well as in the non-primary thalamus and cerebellum, were clearly visible in the time series in awake mice. Among these categories, only the thalamus and cerebellum exhibited notable responses under anesthesia. Direct statistical comparison confirmed broad differences between the groups, with response amplitudes being significantly higher in awake mice in most high-order and cerebellar regions, including PL/IL, Cg, PPC, TeA, PRC, CbH, and CbV (Fig. 3).

A noteworthy observation is that awake animals exhibited a biphasic response in Vis to visual stimulation (Fig. 2), which explains the limited spatial coverage of significant voxels in this region when using the 10-s model in the statistical map analysis (Fig. 1 and Table 1). Similar response shape in awake mice to visual stimulation has been reported previously (Dinh et al. 2021). When a shorter 2-s stimulus block was used in the analysis, significant voxels covered nearly the entire visual cortex (Supplementary Fig. 7), as would be expected.

More interestingly, the time series revealed that cross-sensory responses in the primary cortices (Aud and Vis) displayed temporal profiles distinct from those evoked by sense-specific stimulation. As shown in Fig. 2 (black arrows), the response in Vis to auditory stimulation lacked the later negative component, instead showing a prolonged decay on the positive side. Likewise, in Aud, visual stimulation elicited a slow rise in the signal, whereas auditory stimulation produced a rapid high-amplitude peak (Fig. 2). Together, these observations suggest distinct processing mechanisms in primary cortices for sense-specific versus cross-sensory inputs.

Individual-level average time series for awake and anesthetized experiments are shown in Supplementary Figs. 8 and 9, respectively. In most cases, particularly with bimodal stimuli, the temporal response characteristics were very consistent across mice, indicating high reproducibility across individuals. Notably, cross-sensory and higher-order activities were small but discernible in most of the individual mice, suggesting that while these responses may be reproducible, they remain difficult to detect with limited amount of data. Furthermore, no clear sex-based differences were visible in the time series, as both low- and high-amplitude responses appeared well-mixed across sexes with no clear distinction in their temporal profiles.

### Region-specific response amplitudes to bimodal stimulus do not represent the sum of unimodal responses

Next, we examined whether the fMRI responses to bimodal stimulation were non-additive, being either subadditive or superadditive. Numerical results are provided in Supplementary Table 1 and significant interactions are summarized in Fig. 3. Significant effects were observed in PL/IL, Cg, RSC, Aud, PPC, TeA, LP/DLG, VP/Po, Sub, IC, and CbH. Notably, in all ROIs of awake mice, residuals from the interaction tests were negative (Supplementary Table 1), indicating that responses to bimodal stimulation were predominantly subadditive, i.e. smaller than the sum of responses to unimodal stimuli. Results in anesthetized mice were generally similar, although statistical significance for subadditivity was reached less frequently than in awake mice. Nevertheless, no superadditive interactions were detected in the present work during bimodal stimulation.

Interestingly, several subcortical regions active during visual processing, including PRT, VP/Po, LP/DLG, and SC, showed a trend of smaller responses during bimodal stimulation than during visual stimulation alone (paired *t-*test; *P* = 0.069, uncorrected). In contrast, several cortical polymodal regions active during visual processing, such as TeA, PPC, and PrC, as well as Vis, showed higher response amplitudes during bimodal stimulation compared to visual stimulation alone (paired *t-*test; *P* = 0.009, uncorrected). These observations are well aligned with the differences in the spatial coverage of activation maps (Table 1), in which similar effects were observed in the corresponding regions during bimodal stimulation. Together, these findings suggest distinct cross-sensory effects of auditory stimulation on cortical versus subcortical visual processing.

### Localization of responses within primary and high-order cortices

**Figure 4:**
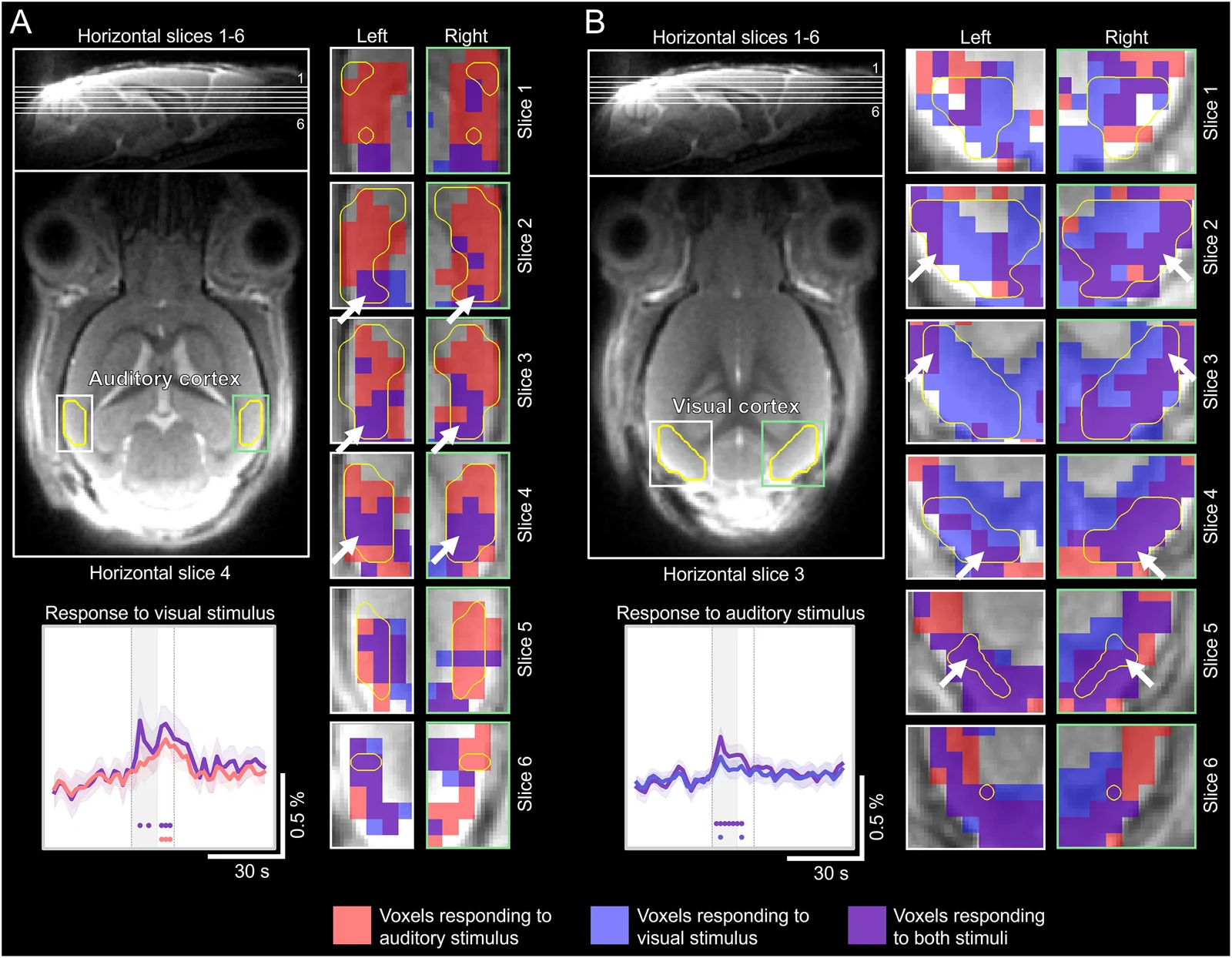
Group-level fMRI responses to unimodal stimuli in auditory (A) and visual (B) cortices of awake mice. Highlighted voxels indicate the presence of significant signal changes (*P* < 0.01, FWE-corrected) to unimodal stimuli. The responses to visual stimulation were analyzed using the shorter 2-s model (see Supplementary Fig. 7), which better reflects the underlying signal. The images on the left of each panel show the localization of each slice. Small white and green boxes indicate the approximate regions from which the zoomed images on the right were obtained. In both panels, six representative horizontal slices are shown, overlaid on an anatomical reference image. Outlines indicate the approximate borders of the primary cortices. Arrows highlight some of the bilateral subregions that responded to both stimulation modalities. Each mean time series is averaged across 100–115 stimuli. The shaded vertical gray region marks the 10-s stimulus block. Gray dashed lines indicate the window for statistical tests. Dots below the time series denote time points that differ significantly from zero (*P* < 0.05, Bonferroni-corrected; one-sample t-test). Shaded areas around the mean curves represent 90% confidence interval.

Finally, we studied the localization of cross-sensory responses to unimodal stimuli in the auditory and visual cortices of awake mice in more detail (Fig. 4). Consistent with the observations above, responses to visual or auditory stimulation covered nearly the entire corresponding cortical areas. In contrast, the cross-sensory responses were more restricted to specific subregions of Aud and Vis. In Aud, the most prominent signal changes evoked by visual stimulation were localized to the dorsal and ventral areas of secondary auditory cortex (belt area), but also covered small regions in the primary auditory cortex (core area). The responses were nearly identical in both hemispheres. In Vis, the strongest responses to auditory stimulation were localized mainly in the lateral area of secondary visual cortex (belt area), but also encompassed small regions of binocular area within primary visual cortex (core area), remaining approximately symmetrical between hemispheres. The analysis of time series revealed that the temporal response profiles clearly differed among voxel groups in Aud, as voxels responsive to both stimulus modalities showed a fast response to cross-modal stimulation, whereas this rapid component was absent in other voxels. In Vis, differences between voxel groups were also present, although the distinctions in temporal signal characteristics were less pronounced. Altogether, these findings support the presence of polymodal subregions within the visual and auditory cortices of awake mice that exhibit stronger responses to cross-sensory input.

In addition to the primary sensory cortices, we investigated whether modality-specific and/or bimodal regions could also be identified in high-order cortical areas. No clear segregation was observed in the PL/IL, Cg, or RSC (data not shown). However, the PPC, PRC, and TeA showed some evidence of modality-specific subregions (Supplementary Fig. 10). In the PPC, visual responses were more pronounced in the anteromedial parts, auditory responses in the lateral parts throughout the region, and bimodal responses in the posterior half. Findings in the PRC and TeA resembled each other: auditory responses were more pronounced in the anterior parts, visual responses in the posterior parts, and bimodal responses in intermediate-posterior parts. Findings were consistent across hemispheres in all regions. These observations suggest that high-order regions may show partial modality-specific subregional organization.

## Discussion

To our knowledge, this is the first fMRI study to demonstrate widespread cross-sensory and high-order activity in the mouse cerebrum and cerebellum in response to auditory and visual stimulation. With both stimulation modalities, subregions showing distinct responses to cross-sensory input were detected in the primary cortices. Consistent with a previous study in rats (Paasonen et al. 2025), anesthesia significantly suppressed cross-sensory and high-order activity during sensory processing. Lastly, simultaneous auditory and visual stimulation evoked fMRI responses that were predominantly subadditive in polymodal regions.

### Brain-wide responses to unimodal auditory and visual stimulation

Despite the mouse being one of the most commonly used model organisms in neuroscience research, the number of fMRI studies investigating auditory (Blazquez Freches et al. 2018; Chen et al. 2020) or visual (Niranjan et al. 2016; Dinh et al. 2021, 2024; Zeng et al. 2022; Hike et al. 2025) systems in mice remains relatively low. Potential reasons include the difficulty of maintaining stable physiological conditions under anesthesia to obtain reproducible and well-localized fMRI responses (Schroeter et al. 2014), as well as the smaller brain size and more pronounced magnetic susceptibility-induced image distortions compared with rats. Recent advances in mouse awake imaging protocols (Chen et al. 2020; Dinh et al. 2021; Zeng et al. 2022; Dvořáková et al. 2024; Hike et al. 2025), along with the implementation of high-field MRI systems (Zeng et al. 2022; Dinh et al. 2024; Hike et al. 2025), have at least partially addressed these challenges. Nevertheless, studies of the auditory system still remain scarce, likely due to the acoustically loud noise of conventional echo planar imaging fMRI techniques (Blazquez Freches et al. 2018), which complicates the experimental setup. These limitations, together with magnetic susceptibility-related image distortions, however, did not affect the current work, as the applied zero-TE method is remarkably quieter and more tolerant of image artifacts than conventional approaches (Paasonen et al. 2020).

The few auditory fMRI studies conducted in mice to date have reported fMRI responses in several parts of the auditory pathways, including Aud, MGB, IC, superior olivary complex, and cochlear nucleus (Blazquez Freches et al. 2018; Chen et al. 2020). In contrast, these studies did not report the brain-wide activity observed in the current work, which includes cross-modal cortices and multiple high-order regions. Notably, however, the activation maps presented by Chen et al. (2020) show evidence of auditory-evoked activity in the visual cortex and non-primary thalamus (Chen et al. 2020), and Blazquez Freches et al. (2018) did observe auditory-evoked activity in the cerebellum, both of which are consistent with our findings and indicative of significant activity outside the primary circuit. In contrast to previous results (Blazquez Freches et al. 2018), we did not observe fMRI responses in the brainstem in the current work. This is likely due to the use of a relatively small MRI surface coil, which provided optimal sensitivity and coverage only to cerebrum and cerebellum.

The visual fMRI studies in mice consistently report signal changes in key visual regions including Vis, DLG, LP, and SC (Niranjan et al. 2016; Dinh et al. 2021, 2024; Zeng et al. 2022; Hike et al. 2025). Additionally, activity in Aud, Cg, PPC, PRC, VP, Po, PRT, and Sub has either been directly reported or is plausible based on the statistical maps presented in these studies. Dinh et al. (2024) further reported positive fMRI signal changes in the somatosensory and motor cortices. Activation of the motor cortex appeared possible in the present study as well, whereas the somatosensory cortex exhibited non-significant negative signal changes, slightly contradicting previous findings. Nevertheless, the remaining results from earlier work (Niranjan et al. 2016; Dinh et al. 2021, 2024; Zeng et al. 2022; Hike et al. 2025) are in very good agreement with our results, strengthening the evidence for brain-wide cross-sensory and high-order activity during visual processing in the mouse brain.

Although many previous individual mouse fMRI studies (Niranjan et al. 2016; Blazquez Freches et al. 2018; Chen et al. 2020; Dinh et al. 2021; Zeng et al. 2022) did not report responses as widespread as those detected in the present work, our observations are supported by the literature. Cg, which exhibited moderate and strong responses to auditory and visual stimuli, respectively, is a high-order region associated with emotion, pain, memory, and attention (Ma et al. 2016; Sun et al. 2022). Cg receives direct input from the visual cortex (Ma et al. 2016), but it also participates in auditory processing (Sun et al. 2022). PL/IL, which also showed moderate and strong responses to auditory and visual stimuli, respectively, is a high-order region involved in motivational and affective behaviors and receives inputs from multiple sources, including both auditory and visual systems (Kringelbach and Rolls 2004). PRC showed responses of roughly similar amplitude to both stimulus types and is known to process polymodal sensory information, including auditory and visual inputs (Furtak et al. 2007; Aminoff et al. 2013). PPC, considered a high-order hub for integrating and relaying somatosensory, auditory, and visual information (Lyamzin and Benucci 2019), exhibited roughly similar responses to both stimulation types in the current work. RSC, another key hub for sensory information flow, exhibits strong connectivity with visual cortices, but there is also evidence of plausible connections with auditory cortices (Alexander et al. 2023), both consistent with our observations of weak and strong responses to auditory and visual stimulation, respectively. Sub is a high-order region that participates in inter-regional communication during spatial information processing and exploratory behavior (Matsumoto et al. 2019). Although Sub does not receive direct sensory inputs, its responses in the current work, mainly to visual stimulation, may be explained by inputs from PRC and RSC (Matsumoto et al. 2019). The exact function of TeA remains uncertain. However, as TeA receives inputs from widespread neocortical regions, including auditory and visual cortices (Zingg et al. 2014), it likely serves an integrative role, which may also account for the strong responses to both stimulation modalities in the present work. The thalamic nuclei VP and Po are traditionally associated with somatosensory processing, but recent evidence indicates that neurons in VP and Po can also be excited by visual stimulation (Allen et al. 2016, 2017), and that VP and Po are susceptible to visual and auditory modulation (Kimura 2024). These cross-sensory interactions may explain the small signal changes observed in the present work within non-primary thalamic regions. Finally, activity in the cerebellar vermis and hemispheres has been reported in response to visual and auditory stimulation, respectively (Baumann and Mattingley 2010; Baumann et al. 2015), which aligns well with our observations. It is important to note that the temporal resolution of the current fMRI data, together with the lag of the hemodynamic response, limits our ability to infer the temporal order of these activities. However, emerging approaches may eventually enable the distinction between bottom-up and top-down activity (Carvalho et al. 2026).

While our observations of brain-wide responses, including high-order regions, non-primary thalamic nuclei, and the cerebellum, appear consistent with structural and electrophysiological evidence, it remains unclear why many previous fMRI studies have reported more restricted activation patterns. One possible explanation is the use of conventional echo planar imaging (EPI) sequences, which can limit spatial coverage (Blazquez Freches et al. 2018), and are prone to image distortions (Chen et al. 2020; Hike et al. 2025) and movement-induced artifacts (Chen et al. 2020), thereby hindering whole-brain and comprehensive data analysis. Another possibility is the traditional research focus on positive responses in a single sensory system, leading to non-primary systems and negative responses being overlooked. Finally, the relatively weak nature of cross-sensory signal changes may make them difficult to detect with limited statistical power, as indicated by our individual-level analyses and previous findings (Paasonen et al. 2025), particularly when overly stringent significance thresholds are applied (Taylor et al. 2023).

### Simultaneous auditory and visual stimulation

Consistent with our observations, simultaneous auditory and visual stimulation evoked subadditive responses in the responding polymodal brain regions in humans (Beauchamp et al. 2004; Martuzzi et al. 2007; Nelson and Mayhew 2025). These findings therefore suggest that multisensory integration exhibits similar characteristics across species. Although individual multisensory neurons can exhibit additive or superadditive responses to multimodal sensory stimulation, subadditive fMRI responses may be explained by the relatively small proportion of such neurons within comparatively large fMRI voxels (Beauchamp et al. 2004). Indeed, studies in macaque monkeys have shown that certain sub-regions in auditory cortices may show clear enhancement during audiovisual stimulus (Kayser et al. 2007), suggesting that the additivity of fMRI signals may be region-dependent and more readily detected in areas with a higher density of superadditive multisensory neurons or with higher spatial resolution. Moreover, some human studies report that both enhancement and attenuation can occur during bimodal stimulation (Baier et al. 2006), implying that the nature and degree of interactions between sensory systems depend on experimental factors such as whether the auditory and visual stimuli are semantically or temporally associated (Baier et al. 2006). Indeed, we also observed that in many regions, bimodal stimulation appeared to suppress the response amplitude to a lower level than that evoked by unimodal stimulation, indicative of attenuation. However, it is important to acknowledge that the potential contribution of hemodynamic response saturation during bimodal stimulation remains poorly understood and may contribute to the observed subadditive responses.

In human studies, the spatial coverage of the bimodal auditory–visual positive response maps resembles the concatenation of unimodal response maps (Martuzzi et al. 2007; Man et al. 2015; Nelson and Mayhew 2025), a finding similar to our study as well. However, we observed multiple subtle differences in the response maps between unimodal and bimodal stimuli, suggesting both spatially expanded and more localized patterns of activity. Notably, bimodal stimulation increased the response coverage in several high-order regions, including PRC, TeA, PPC, and Sub. These may reflect the recruitment of spatially distinct neuronal populations sensitive to specific sensory modalities (Dahl et al. 2009). Whereas responses to bimodal stimulation appeared stronger and more widespread also in the visual cortex, many subcortical regions responding to visual stimulation, including Po, LP/DLG, PrT, and SC, showed slightly more restricted responses to bimodal stimulation. This could be attributed to the inhibitory influences of the bimodal input (Song et al. 2017), potentially occurring when information from different sensory modalities is incongruent or does not provide mutual reinforcement. This explanation aligns with our experimental setup, as the auditory and visual stimuli originated from different spatial locations and had mismatched frequencies.

In our study, we did not observe any regions that showed activity exclusively during bimodal stimulation. A previous study in mice using simultaneous somatosensory and visual stimulation reported widespread thalamic responses that were evident only during bimodal stimulation (Dinh et al. 2024). In humans, some studies using auditory and visual stimulation reported no additional regions activated during bimodal stimulation (Martuzzi et al. 2007), whereas other have shown activation maps suggesting engagement of additional regions under such conditions (Nelson and Mayhew 2025). Thus, the emergence of specific regions in fMRI appears to depend on the particular combination of sensory stimuli or other experimental conditions, as also discussed earlier (Calvert 2001).

### Responses within auditory, visual, and high-order cortices

Given that primary auditory and visual cortices are increasingly regarded as multisensory brain regions across species (Choi et al. 2023), the fMRI responses to cross-modal stimulation observed here are consistent with this view. Consistent with our findings, studies in humans (Martuzzi et al. 2007; Raij et al. 2010; Man et al. 2015; Gu et al. 2020) and non-human primates (Kayser et al. 2007; Opoku-Baah et al. 2021) have reported robust responses to visual stimulation in Aud. Similarly, supporting evidence for auditory stimulation-evoked activity in Vis has been reported in humans (Martuzzi et al. 2007; Raij et al. 2010; Man et al. 2015), cats (Ghazanfar and Schroeder 2006; Petro et al. 2017), and blind rats (Maruoka et al. 2024). However, while cross-sensory responses to auditory stimulation were clearly visible with magnetoencephalography and optical imaging, they were not detected with fMRI in few studies (Raij et al. 2010; Gu et al. 2020), likely because of the loud acoustic noise of the EPI sequence (Raij et al. 2010).

The cross-sensory responses to auditory and visual stimulation observed in the current work were localized to both primary and secondary cortices. Although previous human studies have primarily reported responses in the primary cortices (Martuzzi et al. 2007; Raij et al. 2010; Man et al. 2015), the border (or belt) areas are known to contain high density of multisensory neurons (Ghazanfar and Schroeder 2006). In many earlier human studies, the reported responses were relatively widespread, likely encompassing secondary cortices as well. Moreover, studies in macaque monkeys have explicitly demonstrated cross-sensory activity in both primary and secondary sensory areas (Kayser et al. 2007), further supporting our observations and highlighting the cross-species similarity in the localization of cross-sensory processing.

Although the auditory and visual systems are among the most extensively studied from multisensory perspective (Opoku-Baah et al. 2021), considerable variability exists across the results. One of the most inconsistent observations concerns whether unimodal sensory stimulation evokes a positive or negative hemodynamic response in cross-modal brain regions. While our findings show mainly positive cross-sensory responses to unimodal stimulation, similar to many human studies (Martuzzi et al. 2007; Raij et al. 2010; Man et al. 2015; Gu et al. 2020), other human studies reported clear negative cross-sensory responses (Laurienti et al. 2002; Nelson and Mayhew 2025), interpreted as deactivations. Although more technical explanations, such as scanner noise-induced baseline modulation or differences in post-processing, have been speculated (Laurienti et al. 2002), it is likely that variability in response polarity depends on whether the attention is directed to one or both stimulation modalities (Calvert 2001; Martuzzi et al. 2007). For example, when attention is focused on one modality, the brain may suppress distracting sensory input from the other. Future studies with well-controlled attention paradigms are therefore needed to further understand these interactions.

In addition to localized cross-sensory processing in primary cortices, our data support the idea that some high-order cortical areas, such as the PPC, PRC, and TeA, may exhibit a slight bias toward region-specific processing of different sensory modalities. Previous studies have shown that parietal, temporal, and perirhinal areas may contain small neuronal populations or more defined subregions that preferentially respond to auditory versus visual information, or vice versa (Bushara et al. 1999; Xu et al. 2019; Chai et al. 2021; Lim and Lee 2024), consistent with our observations. These patterns may reflect graded hierarchical organization and differential connectivity with modality-dominant cortical and subcortical inputs. Notably, several of the previous studies were conducted using human neuroimaging, raising the possibility that similar features may be present across species and detectable with imaging methods.

### The confounding effects of anesthesia during auditory and visual cross-sensory and high-order processing

To our knowledge, no whole-brain studies have examined the effects of anesthesia on cross-sensory and high-order responses to auditory or visual stimulation. In a study focusing on Aud of macaque monkeys, Kayser et al. (2007) showed that remifentanil anesthesia restricts the visual stimulation-evoked cross-sensory responses. In our work, the confounding effect of anesthesia was even more pronounced, as we did not observe any activity in Aud under ketamine-xylazine anesthesia, whereas this region responded robustly to both stimulus modalities in awake mice. However, despite the absence of responses in Aud, our whole-brain approach revealed that visual stimulation elicited detectable responses in several high-order areas and non-primary thalamic regions in anesthetized mice. These findings, together with previous work under similar experimental conditions (Dinh et al. 2024), suggest that many aspects of brain-wide high-order processing can be evoked by visual stimulation even in anesthetized mice. Nevertheless, responses in most regions were significantly more restricted compared to those obtained in awake mice, and cross-modal activity in primary sensory cortices was not observed.

Outside the auditory areas, we observed robust responses to auditory stimulation only in the cerebellum under ketamine-xylazine anesthesia, indicating a widespread absence of activity in visual and high-order areas. This likely reflects strong suppression of the primary sensory pathway, as both the auditory thalamus and cortex showed no detectable responses. The only clear exception was IC, which is known for its ability to relay both auditory and visual information (Gruters and Groh 2012). As discussed earlier, ketamine may affect the auditory system more strongly than the visual system (Dvořáková et al. 2024), suggesting that this compound may be unsuitable for auditory studies. Supporting evidence for this has also been reported in marmosets (Dureux et al. 2025). Taken together, ketamine-xylazine anesthesia appears to have a detrimental impact on primary, cross-sensory, and high-order auditory processing.

Compared to the awake state, the general suppression of responses in high-order regions such as Cg, PL/IL, PRC, TeA, and PPC under anesthesia is expected, as disruption of integrative processing is among the proposed mechanisms of anesthetic action (Montupil et al. 2023). However, because anesthetics exert unique effects on brain organization (Paasonen et al. 2018), it is likely that the outcomes of the present study are anesthetic-dependent. A previous study in rats studying cross-sensory and high-order activity in response to somatosensory stimulation reported a suppression of high-order processing under isoflurane-medetomidine anesthesia (Paasonen et al. 2025), similar to the current findings. In contrast, cross-sensory responses, although limited, were observed across multiple sensory systems under anesthesia, extending to the primary cortices (Paasonen et al. 2025). Therefore, while suppression of high-order activity appears to be a common feature, the specific combination of anesthesia protocol and stimulus modality may influence the extent of cross-sensory processing.

### Limitations

Several measures were implemented to minimize the effect of motion on the fMRI data. First, animals were carefully habituated to the imaging procedure. Second, we employed a quiet and movement-tolerant zero-TE fMRI sequence (Paasonen et al. 2020). Third, the skull was firmly secured to the imaging holder, effectively eliminating the brain movement. Fourth, the data were motion-corrected, and the resulting motion parameters were included as nuisance regressors in the analysis. A detailed description of these have been reported previously (Dvořáková et al. 2024). Nevertheless, we cannot completely rule out potential confounding effects of movement on our results.

The spatial resolution of the fMRI data in the present study (375 μm, isotropic) was not sufficient to define highly detailed ROIs that would accurately isolate specific subregions within brain structures. This limitation likely reduced the sensitivity of the ROI-based analyses, as cross-sensory activity was often confined to only a portion of the anatomically defined, larger ROIs. In addition, partial volume effects and point spread function make it difficult to localize activations precisely at border regions. Because each fMRI voxel encompasses multiple neuronal populations, identifying the exact source of the observed cross-sensory responses with fMRI is not possible. Similarly, because the fMRI signal reflects net neural activity, it is difficult to assess the contributions of concurrent changes in arousal, vigilance, and/or stress during neural responses to sensory stimulation. Therefore, signals observed particularly in higher-order regions may partially reflect more general physiological state changes.

Neurovascular coupling and its linearity may vary across the thalamic and cortical areas investigated and under different stimulus conditions. Nevertheless, we still assume that region-specific changes in the fMRI signal qualitatively reflect changes in the underlying neuronal activity, allowing us to interpret whether one stimulus condition evokes more or less neuronal activity than another within a given region. However, comparisons of response amplitudes across different brain regions should be interpreted with caution.

Because bilateral stimulation was used in the present study, conclusions about the laterality of cross-sensory and high-order processing cannot be made.

During habituation and imaging, mice were exposed to isoflurane anesthesia multiple times. Although we observed no major evidence of lingering effects of isoflurane on the spatial response patterns, isoflurane has been shown to exert long-term effects on brain function (Stenroos et al. 2021). Therefore, its potential influence on the present findings cannot be fully excluded.

Finally, although zero-TE fMRI signal correlates well with neuronal activity (Valjakka et al. 2025), the physiological basis of its functional contrast is not yet fully characterized. Our current understanding is that the contrast primarily originates from activation-induced increases in cerebral blood volume (CBV) and cerebral blood flow (CBF), which introduce greater amounts of unsaturated water magnetization into the voxel, with a potentially smaller contribution from changes in tissue oxygenation (Mangia et al. 2026). This interpretation is supported by the sensitivity of the functional contrast to flip angle (Lehto et al. 2017) and by the close correspondence between the zero-TE hemodynamic response function and those of CBV and CBF (Valjakka et al. 2025). Therefore, the zero-TE findings should be comparable to those obtained with CBF- and CBV-based methods. However, the majority of previous sensory studies have used blood oxygenation level-dependent (BOLD) contrast, which differs fundamentally from CBV- and CBF-based approaches. Whereas changes in CBF and CBV primarily occur on the arterial and capillary sides of the vasculature, relatively close to the site of neuronal activation, BOLD responses are more weighted toward large draining veins, which may be located at some distance from the activation site (Buxton 2013). In addition, CBF and CBV may exhibit a different dynamic range because BOLD contrast is subject to a ceiling effect, and the linearity of responses across different stimulation intensities may differ between these contrasts (Buxton 2013). Despite these differences, both CBF/CBV and BOLD contrasts rely on neurovascular coupling and are expected to provide qualitatively similar results reflecting neural activity. Yet, subtle differences in signal amplitudes and the spatial localization of vascular signals may contribute to discrepancies between the present findings and previous BOLD-based studies.

## Supplementary Material

Supplementary_material_bhag137

## Data Availability

The data used here is included in the data package openly available at https://doi.org/10.23729/f25d01ae-1b27-4395-8446-2dfd37d80a76.
