# Supplementary material for: Brain-wide fMRI responses to auditory and visual stimuli in awake mice
Source: Cereb Cortex. 2026 Sep 16;36(9):bhag137. doi: 10.1093/cercor/bhag137 (PMC13580225; doi:10.1093/cercor/bhag137)
Supplement: Supplementary file 1 [file supplementary_material_bhag137.zip › Supplementary_Material_bhag237.docx]

**Supplementary Materials for “Brain-wide fMRI responses to auditory and visual stimuli in awake mice”**

Jaakko Paasonen^1,*^, Lenka Dvořáková^1^, Petteri Stenroos^1^, Raimo A. Salo^1^, Ekaterina Paasonen^1,2^, Shalom Michaeli^3^, Silvia Mangia^3^, Heikki Tanila^1^, Olli Gröhn^1^

^1^ A. I. Virtanen Institute for Molecular Sciences, University of Eastern Finland, Kuopio, Finland
^2^ NeuroCenter, Kuopio University Hospital, Kuopio, Finland
^3^ Center for Magnetic Resonance Research, University of Minnesota, Minneapolis, United States

*Corresponding author. A.I.V. Institute for Molecular Sciences, University of Eastern Finland, P.O. Box 1627, FI-70211, Kuopio, Finland. Phone number: +358403552219. E-mail address: jaakko.paasonen@uef.fi

**Supplementary Figure 1. Schematic illustration of the visual (A) and auditory (B) stimulation setups, the stimulation blocks (C), and details of the repeated blue light flashes and air puffs used to induce visual and auditory sensations, respectively.** Blue light (470 nm) was delivered to both eyes through optical fibers at an intensity of 41 lx. Air puffs were delivered through an open tube positioned 15 cm posterior to the mouse's head. The average sound pressure levels measured at the level of the mouse were 69 dB and 82 dB during the fMRI sequence and during the fMRI sequence with stimulation, respectively. The frequency spectrum of the air puffs spanned a wide range of frequencies, resembling white noise. The order of stimulus presentation was randomized independently for each experiment. Partially created with BioRender.com

**Supplementary Figure 2. Anatomical template (top row) and functional image from a representative mouse (bottom row) shown in three different orientations.** Both images were acquired with 3D MB-SWIFT sequence with isotropic resolutions of 94 µm (anatomical) and 375 µm (functional). Anatomical template is an average across all data sets in the study.

**Supplementary Figure 3. Three-dimensional presentation of clusters showing significant signal changes (p < 0.01, FWE-corrected) in response to auditory, visual, and bimodal stimuli in awake and anesthetized mice (A), along with the selected regions-of-interests (ROIs) (B).** Results in A are based on 100-115 stimulus blocks acquired across 20–23 scans per group. ROI color coding in B corresponds to that in Figure 1. Supplementary Video 1 shows the same data (A and B) from different viewing angles around the brain.

**Supplementary Figure 4.** **Statistical map reproducibility and regions of interest (ROIs) across individual awake and anesthetized mice.** Incidence maps summarize voxel-level signal change reproducibility across individual mice responding to auditory, visual, and bimodal stimuli. To generate these maps, a statistical activation map was first calculated for each mouse. Individual maps were then thresholded at t > 2, binarized, and summed. Voxels present in only one animal were excluded. The resulting incidence maps display the percentage of subjects exhibiting t > 2 within each voxel. Every other coronal slice is shown, overlaid on an anatomical reference image. Aud, auditory cortex; CbH, cerebellar hemisphere; CbV, cerebellar vermis; Cg, cingulate cortex; DLG, dorsal lateral geniculate nuclei; IC, inferior colliculus; IL, infralimbic cortex; Ket+Xyl, ketamine-xylazine anesthesia; LP, lateral posterior thalamic nuclei; MGB, medial geniculate body; PL, prelimbic cortex; Po, posterior thalamic complex; PPC, posterior parietal cortex; PRC, perirhinal cortex; PrT, pretectal area; RSC, retrosplenial cortex; SC, superior colliculus; Sub, subicular complex; TeA, temporal association cortex; Vis, visual cortex; VP, ventral posterior thalamic complex.

**Supplementary Figure 5.** **Sex-specific response reproducibility across awake and anesthetized states.** Incidence maps summarize voxel-level signal change reproducibility across male (n = 4) and female (n = 3-4) mice responding to auditory, visual, and bimodal stimuli. To generate these maps, a statistical activation map was first calculated for each mouse. Voxels present in only one animal were excluded. Individual maps were then grouped by sex, thresholded at t > 2, binarized, and summed. The resulting incidence maps display the percentage of subjects exhibiting t > 2 within each voxel. Every fourth coronal slice is shown, overlaid on an anatomical reference image.

**Supplementary Figure 6. Group-level differences (p < 0.01, FWE-corrected) between statistical maps generated from the first five and last five stimulation blocks during bimodal stimulation in awake mice.** Significant differences were limited to a single voxel in the border of hypothalamus and a two-voxel cluster in the visual cortex. No significant differences were observed for either unimodal stimulus. Similar results were obtained using lower statistical thresholds (p < 0.05 and p < 0.1; data not shown). Statistical maps are overlaid on anatomical images.

**Supplementary Figure 7. Activation maps in response to auditory and visual stimuli in awake mice, along with selected regions-of-interest.** For this analysis, the stimulus duration was set to 2 s to match the positive phase of the visual response. White outlines on the maps indicate regions with p < 0.01 (FWE-corrected), and color maps are thresholded at t > 2.5. Results are based on 115 stimulus blocks collected across 23 scans. Four selected coronal slices are shown, overlaid on an anatomical reference image. Aud, auditory cortex; DLG, dorsal lateral geniculate nuclei; IC, inferior colliculus; LP, lateral posterior thalamic nuclei; MGB, medial geniculate body; PPC, posterior parietal cortex; PRC, perirhinal cortex; PrT, pretectal area; RSC, retrosplenial cortex; SC, superior colliculus; Sub, subicular complex; TeA, temporal association cortex; Vis, visual cortex.

**Supplementary Figure 8. Individual-level mean time series from regions-of-interest (ROIs) in awake mice (n = 8).** The shaded vertical gray region marks the 10-s stimulus block. Gray lines indicate the window for calculating average responses. Abbreviations for ROIs are provided in Supplementary Figure 1, Figure 1, and in Table 1.

**Supplementary Figure 9. Individual-level mean time series from regions-of-interest (ROIs) in anesthetized mice (n = 7).** The shaded vertical gray region marks the 10-s stimulus block. Gray lines indicate the window for calculating average responses. Abbreviations for ROIs are provided in Supplementary Figure 1, Figure 1, and in Table 1. Ket+Xyl, ketamine-xylazine anesthesia.

**Supplementary Figure 10. Group-level fMRI responses to unimodal stimuli in the posterior parietal cortex (PPC; A), temporal association cortex (TeA; B), and perirhinal cortex (PRC; C) of awake mice.** Color-coded voxels indicate significant signal changes (p < 0.01, FWE-corrected) in response to unimodal stimuli. Each panel shows representative 6–7 slices covering the corresponding region, with whole-slice views shown in the bottom row and zoomed views of the corresponding region in both hemispheres shown in the top row. Yellow lines indicate the approximate borders of the corresponding region. White lines overlaid on the sagittal anatomical images on the right indicate the locations of the slices.

**Supplementary Table 1. Residuals and p-values from the interaction tests for each region-of-interest.** Outliers were removed prior testing by using the Q1 - 1.5 IQR and Q3 + 1.5 IQR thresholds. Negative residual indicates that the bimodal response amplitude was lower compared to the sum of unimodal response amplitudes. P-values are uncorrected.
